# How can mental health and substance use services become dual diagnosis capable? Moving from theory into practice

**DOI:** 10.1186/1472-6963-14-S2-P116

**Published:** 2014-07-07

**Authors:** Petra Staiger, Alexandra Howard, Anna C Thomas, Greg Young, Marita McCabe

**Affiliations:** 1 School of Psychology, Deakin University, Burwood, Victoria, Australia; 2 Australian Institute of Family Studies, Victoria, Australia; 3 Southern Health Care Network, Clayton, Victoria, Australia

## Background

There is evidence that separate service delivery for individuals with dual Mental Health and Substance Use Disorders is expensive and can have negative treatment implications. While redesigning the health system to provide integrative care is optimal, costs of this mean that the focus has generally been on developing models of care and best practice guidelines to improve the identification and treatment of dual diagnosis within current systems. ‘Shared care’ with MH and SUD services collaborating to coordinate delivery of interventions is considered the best option within these parameters. However, only a few projects have reported on the implementation of such highly collaborative models and many of these have not yet been systematically evaluated. The current study represents the implementation and evaluation of an Early Identification Model (EIM) in three health services settings. The EIM was created to support health services through the complex dynamics of becoming dual diagnosis capable. It consists of two primary components: two-step systematic screening, and clearly defined care pathways to ensure that dual diagnosis identification leads to appropriate follow up, referral, or delivery of evidence-based interventions for each individual.

## Methods

The model was implemented at three health service sites (mental health; alcohol and drug; community health). The methodology consisted of three components. First, the literature on dual diagnosis and health service systems was consulted; second, the findings of the evaluations (i.e., focus group data and quantitative findings) were reviewed from the three sites; and, finally, feedback from an expert advisory group who consisted of senior academics and clinicians in the field was incorporated.

## Results

The four principles arrived at were to: (a) embed the model into existing systems; (b) establish clear guidelines for clinical decisionmaking and care pathways; (c) use a change management framework during the implementation phase; and (d) provide training and capacity building to staff.

## Conclusions

Although the three services were able to successfully implement the new screening and referral pathway procedures with their available resources, it was clear that additional funding would be required in the longer term to accommodate more integrated in-house treatment of clients with a dual diagnosis. SUD staff in particular felt they did not have the required level of skills to treat MH issues so further training in mental health management would be useful in the longer term. Investment in training staff is likely to substantially improve client outcomes without the need for immediate structural change in the current health care system.

